# Evaluation of Imatinib and Its Combination With Albendazole Against *Echinococcus granulosus* Protoscoleces and Microcysts In Vitro

**DOI:** 10.1155/japr/6521300

**Published:** 2026-06-30

**Authors:** Zahra Hanifeh, Ali Haniloo, Negin Torabi, Mehdi Farhadi

**Affiliations:** ^1^ Department of Parasitology and Mycology, School of Medicine, Zanjan University of Medical Sciences, Zanjan, Iran, zums.ac.ir

**Keywords:** albendazole, *Echinococcus granulosus*, hydatid cyst, imatinib, microcyst, protoscoleces

## Abstract

**Background:**

Currently, benzimidazoles are the main drugs used for the treatment of hydatidosis. However, the available medications do not demonstrate the expected therapeutic efficacy. As a tyrosine kinase inhibitor, imatinib may interfere with parasite signaling pathways and exert antiparasitic effects. The present study is among the first to evaluate imatinib in *Echinococcus granulosus*, especially on microcysts and in combination with albendazole. The aim of this study was to investigate the in vitro antiparasitic effects of imatinib and its combination with albendazole on the protoscoleces and microcysts of *E*. *granulosus*.

**Materials and Methods:**

The effects of imatinib alone and in combination with albendazole were assessed on protoscoleces and microcysts over 33 and 15 days, respectively, and compared with albendazole monotherapy. The viability percentage of protoscoleces was determined using the eosin exclusion assay. Ultrastructural and morphological changes were evaluated using scanning electron microscopy and inverted microscopy.

**Results:**

Maximum protoscolicidal activity was observed at a concentration of 40 *μ*g/mL imatinib and the combination of 20 *μ*g/mL albendazole + 20 *μ*g/mL imatinib, both of which resulted in complete mortality of protoscoleces within 6 days. In contrast, 6 days after exposure to 10 *μ*g/mL albendazole, 84.33*%* ± 4.5*%* of protoscoleces remained viable. Exposure of microcysts to 15 *μ*g/mL imatinib and the combination of 10 *μ*g/mL albendazole + 1 *μ*g/mL imatinib led to their complete destruction by Day 6.

**Conclusion:**

The findings of this study demonstrated that imatinib has a significant effect on the protoscoleces and larval cyst forms of *E*. *granulosus*, showing higher protoscolicidal activity compared with albendazole. Moreover, the combination of imatinib and albendazole exhibited stronger and faster effects than monotherapy. These effects may be related to inhibition of tyrosine kinase–dependent pathways. Further in vivo studies are required to confirm these results and evaluate their potential clinical implications.

## 1. Introduction

Cystic echinococcosis (CE) is a highly significant zoonotic parasitic disease of medical and veterinary importance, caused by the larval stage (metacestode) of *Echinococcus granulosus*, a cestode belonging to the Taeniidae family. Epidemiologically, CE has a global distribution and is prevalent in many regions, especially in endemic areas including parts of Asia, South America, and the Middle East, where it is considered a major public health problem, with an estimated incidence of > 50 per 100,000 person‐years and around 1 million people affected worldwide [1]. It is characterized by unilocular, fluid‐filled cysts that develop in different organs such as the liver, lungs, and even the brain. The life cycle of the parasite includes definitive hosts, mainly canids, and intermediate hosts such as herbivores, and humans act as accidental hosts. Infection occurs through ingestion of the worm′s eggs, leading to the formation of hydatid cysts in target organs [2, 3].

Given the clinical consequences of cyst development, effective management strategies are essential. The main therapeutic approaches for hydatid cysts include surgical removal of the cyst, percutaneous treatments such as the PAIR, and chemotherapeutic management using benzimidazoles. Albendazole, the first‐line drug, is administered orally and is commonly used before and after invasive procedures to reduce the viability of protoscoleces. In some cases—particularly when the cyst is small or surgery is not feasible—active monitoring (“watch‐and‐wait”) is also considered as a management option [4]. Currently, the only pharmacological agents used for CE treatment are benzimidazoles such as albendazole and mebendazole. However, none of these medications demonstrate the expected therapeutic efficacy, and they fail to effectively cure the disease. Albendazole is the drug of choice for preventing secondary cyst formation during surgery and for treating inoperable hydatid cysts. Nevertheless, despite high daily doses and long treatment durations (up to 6 months), the success rate of therapy is only about 20%–50% [1, 5, 6]. Furthermore, several adverse effects of albendazole—including encephalitis, drug‐induced rashes, leukopenia, and allergic purpura—have been reported in some patients [1]. These limitations highlight the need to examine alternative or complementary therapeutic agents. Given the lengthy and costly process of developing new antiparasitic drugs [7], optimizing existing medications and evaluating alternative antiparasitic agents or novel compounds for the treatment of hydatid disease is urgently needed.

Imatinib, commercially known as Gleevec, is an oral drug belonging to the 2‐phenylaminopyrimidine family and is commonly used in the treatment of certain cancers. This small molecule functions as a tyrosine kinase inhibitor and targets receptors such as ABL, c‐KIT, PDGFR‐*β*, as well as others including CSF1R and FLT3. Clinically, imatinib is administered for the management of chronic myeloid leukemia (CML), certain cases of acute lymphoblastic leukemia (ALL), gastrointestinal stromal tumors, hypereosinophilic syndromes, and chronic eosinophilic leukemia [8–10]. Imatinib acts by inhibiting tyrosine kinase activity and thereby interferes with key signaling pathways involved in cell proliferation and survival [11]. Recent studies have demonstrated that imatinib possesses antiparasitic properties in addition to its anticancer effects. Imatinib inhibits growth and induces death of *Schistosoma mansoni* in a manner dependent on both exposure time and dose [12]. Investigation of the effects of imatinib on the larval stage of *Echinococcus multilocularis* revealed that imatinib, at 25 *μ*M in vitro, is markedly effective in killing *Echinococcus* protoscoleces, metacestode vesicles, and stem cells. Enzymes with strong homology to human ABL tyrosine kinases have been identified in *Echinococcus*, suggesting a similar molecular target. Notably, preliminary studies indicate that, at concentrations used for parasite culture, imatinib exerts minimal effects on human cells, further supporting its potential as a credible replacement for benzimidazoles in anti‐*Echinococcus* chemotherapy [13]. However, the effects of imatinib on *E. granulosus*, especially in combination with albendazole, remain insufficiently explored. Therefore, this study is aimed at evaluating the in vitro effects of imatinib alone and in combination with albendazole, compared with albendazole monotherapy, against *E. granulosus* protoscoleces and microcysts.

## 2. Methods

### 2.1. Collection of Protoscoleces and In Vitro Incubation Procedure

In 2024, *E. granulosus* protoscoleces were collected under sterile conditions from hydatid cysts located in the livers of infected sheep slaughtered in the slaughterhouses of Zanjan province. Multiple fertile cysts were used to obtain the required number of protoscoleces for the experiments. Calcified or microbially contaminated cysts were excluded from the study. Hydatid fluid from intact and noninfected cysts was aspirated using a sterile syringe near a flame and transferred into sterile Falcon tubes. The protoscoleces were rinsed several times with phosphate‐buffered saline solution supplemented with penicillin and streptomycin, using centrifugation at 1500 rpm for 5 min. Body movements and activity of flame cells in the protoscoleces were observed through a light microscope, and their viability was subsequently determined using the 0.1% eosin exclusion test.

To evaluate the effects of the drugs, stock solutions of imatinib and albendazole at a concentration of 1000 *μ*g/mL were prepared by dissolving imatinib powder in distilled water and albendazole in DMSO. Albendazole was kindly provided as a gift by Kimia Salamat Khavaremianeh Pharmaceutical Company (Iran), and imatinib was purchased from Sobhan Oncology Company (Iran). Protoscoleces demonstrating more than 95% viability were placed into culture flasks containing 10 mL of RPMI medium enriched with 100 *μ*g/mL streptomycin and 100 U/mL penicillin (2000 protoscoleces per culture flask). The protoscoleces were then exposed to imatinib at final concentrations of 1, 2.5, 5, 10, 20, and 40 *μ*g/mL. In addition, they were treated with combinations of albendazole and imatinib at concentrations of 20 *μ*g/mL albendazole + 20 *μ*g/mL imatinib, 10 *μ*g/mL albendazole + 10 *μ*g/mL imatinib, and 5 *μ*g/mL albendazole + 5 *μ*g/mL imatinib. One culture flask containing 100 *μ*L of DMSO (final concentration: 1% *v*/*v*) and another containing distilled water were used as negative controls. The culture flasks were incubated without medium replacement at 37°C and 5% CO_2_ for 33 days. The experiment was conducted in triplicate. Throughout the incubation period, samples containing approximately 70 protoscoleces were collected every 3 days from each group using a micropipette, and their viability was evaluated using a 0.1% eosin dye exclusion assay.

To examine the ultrastructural alterations induced in the protoscoleces, samples from the treated and control groups were collected on the tenth day of incubation and prepared for scanning electron microscopy (SEM) according to the method outlined by Elissondo et al. [14]. A total of 300 protoscoleces per group were analyzed under SEM. Shortly thereafter, the parasites were rinsed with a sodium cacodylate buffer and immersed in glutaraldehyde (2.5%) for 24 h at 4°C. The specimens were then subjected to three rounds of buffer rinsing using centrifugation at 1500 rpm for 5 min. Subsequently, the protoscoleces were placed on poly‐L‐lysine–coated coverslips and dehydrated through a graded ethanol series (50%–100%). For final drying, the specimens were placed into a mixture of hexamethyldisilazane and absolute ethanol for 20 min, followed by immersion in a 2:1 solution of hexamethyldisilazane and ethanol for another 20 min, and finally incubated in pure hexamethyldisilazane for 45 min. The dried samples were then coated with a thin layer of gold and examined using a SEM (Rastak Co.) operated at 25kV.

### 2.2. Formation of Microcysts and Their In Vitro Incubation Process

The transformation of *E. granulosus* protoscoleces into microcysts was carried out using the vesicular culture method under in vitro conditions [15]. Under sterile conditions, viable protoscoleces (> 95%) were washed with PBS. The protoscoleces were then cultured in flasks containing 20 mL of RPMI culture medium enriched with 20% (*v*/*v*) fetal calf serum, 4 mg/mL D‐glucose, 100 *μ*g/mL streptomycin, and 100 U/mL penicillin. The flasks were kept at 37°C in a 5% CO_2_ atmosphere for 60 days, and the medium was refreshed every 4 days. The formed microcysts were collected from the flasks using a Pasteur pipette and allocated into seven treatment groups and two positive control groups (10–12 microcysts per group). The microcysts were then treated separately with imatinib at final concentrations of 1, 2.5, 5, 10, and 15 *μ*g/mL, as well as with combinations of 10 *μ*g/mL albendazole + 1 *μ*g/mL imatinib and 5 *μ*g/mL albendazole + 1 *μ*g/mL imatinib. A 1% DMSO solution was used as the negative control. The flasks containing microcysts were incubated for 15 days at 37°C in a 5% CO_2_ atmosphere. The experiment was performed in triplicate. Microcysts were examined daily under an inverted light microscope to evaluate morphological alterations and structural destruction, based on predefined criteria including loss of turgidity, collapse, and degeneration of the cyst wall.

### 2.3. Statistical Analysis

The experimental results were expressed as mean ± standard deviation (SD). Data were analyzed statistically with SPSS (Version 27). A one‐way ANOVA followed by Bonferroni post hoc test was used to assess how different drug treatments influenced the viability of protoscoleces and microcysts. In all analyses, a *p* value < 0.05 was considered significant.

## 3. Results

### 3.1. Effects of Imatinib and the Combination of Imatinib and Albendazole on *E. granulosus* Protoscoleces

**Figure 1:**
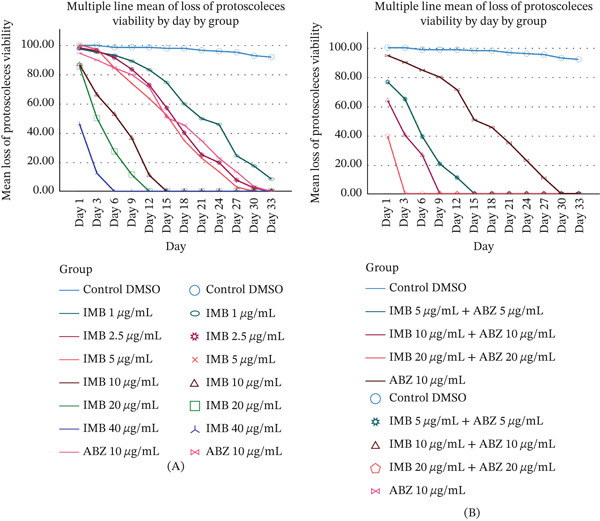
Viability of protoscoleces under in vitro conditions during exposure to (A) imatinib (IMB) alone and (B) the combination of IMB and albendazole (ABZ). Both 2.5 *μ*g/mL imatinib and 10 *μ*g/mL albendazole caused complete protoscoleces′ death by Day 33. Each point shows the mean proportion of viable protoscoleces across days, obtained from three independent experimental replicates (all experiments performed in triplicate).

The viability of *E. granulosus* protoscoleces during exposure to different concentrations of imatinib and the combination of imatinib and albendazole is presented in Figure 1.

In this study, the effects of imatinib alone at concentrations of 1, 2.5, 5, 10, 20, and 40 *μ*g/mL, as well as in combination with albendazole at concentrations of 20 *μ*g/mL albendazole + 20 *μ*g/mL imatinib, 10 *μ*g/mL albendazole + 10 *μ*g/mL imatinib, and 5 *μ*g/mL albendazole + 5 *μ*g/mL imatinib, were evaluated on protoscoleces in comparison with albendazole alone. Drug concentration and incubation time showed a direct relationship with mortality, indicating that both factors jointly influence protoscoleces′ death under in vitro conditions. The viability of protoscoleces on Day 33 in the negative control (DMSO) group was 84.53*%* ± 2.72*%*.

Comparison among the different groups showed that the highest protoscoleces′ mortality was observed with 40 *μ*g/mL imatinib and the combination of 20 *μ*g/mL albendazole + 20 *μ*g/mL imatinib, both causing complete death of protoscoleces by Day 6. Notably, the combination of 5 *μ*g/mL albendazole + 5 *μ*g/mL imatinib, which caused complete protoscoleces death by Day 12, was significantly more effective compared with the effects of 10 *μ*g/mL imatinib alone (89% mortality on Day 12) and 10 *μ*g/mL albendazole alone (29% mortality on Day 12) (*p* < 0.05). These results suggest that the highest efficacy in the combination treatments was primarily attributable to imatinib, whereas albendazole appeared to play a minor role.

The lowest efficacy of imatinib was observed at 1 and 2.5 *μ*g/mL, with 91.66% and 100% protoscoleces mortality by Day 33, respectively.

After 15 days of exposure to 10 *μ*g/mL imatinib, all protoscoleces were killed, whereas 50.33*%* ± 8.5*%* of protoscoleces exposed to 10 *μ*g/mL albendazole were still viable at the same time point, with complete death occurring only by Day 33 (*p* < 0.05). A comparison of 5 *μ*g/mL imatinib with 10 *μ*g/mL albendazole also showed a trend toward higher efficacy for imatinib, with complete protoscoleces death occurring on Days 30 and 33, respectively (*p* > 0.05).

**Figure 2:**
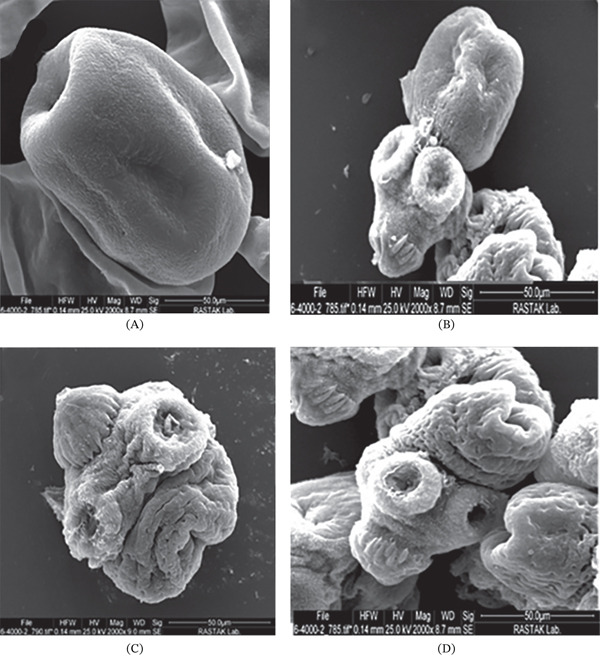
SEM images of *Echinococcus granulosus* protoscoleces. (A) Control protoscoleces after 10 days of treatment. (B) Loss of several hooks and deformation of the rostellum in protoscoleces treated with 10 *μ*g/mL imatinib. (C) Contraction of the soma region in protoscoleces treated with 10 *μ*g/mL imatinib. (D) Damage to the suckers, tegument, and microtriches in protoscoleces treated with 10 *μ*g/mL imatinib.

The results from SEM revealed that protoscoleces in the control group showed no noticeable morphological or ultrastructural changes. In contrast, ultrastructural damage was evident in protoscoleces exposed to imatinib. SEM analysis demonstrated that 10 days after treatment with 10 *μ*g/mL imatinib, protoscoleces exhibited rostellum deformation, a noticeable reduction in hooks, shedding of microtriches around the scolex, contraction of the soma, and ultimately tegumental damage (Figure 2).

### 3.2. Effects of Imatinib on *E. granulosus* Microcysts

After culturing the protoscoleces in glucose‐ and FBS‐enriched RPMI medium for 2 months, the budding protoscoleces, approximately 100 *μ*m in size, gradually transformed into *E. granulosus* microcysts. The cysts clearly increased in size, became swollen, and over time, as their diameter increased, the thickness of their walls also increased. These mature microcysts were used as an appropriate model to assess the effects of drugs on the metacestode stage of the parasite.

**Figure 3:**
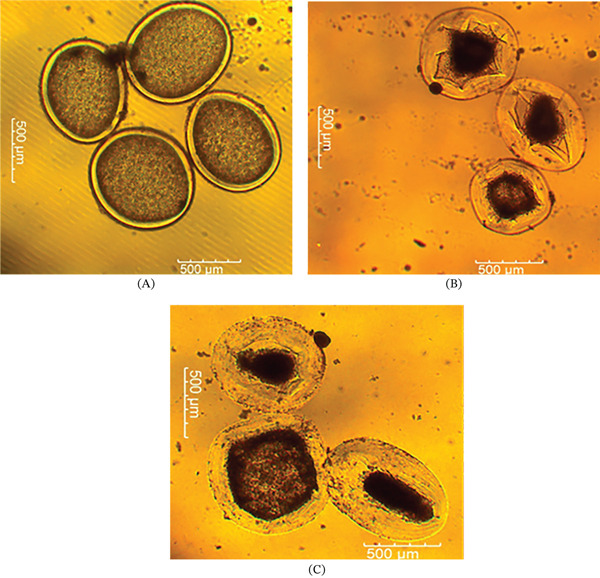
Inverted microscope images of *Echinococcus granulosus* microcysts. (A) Swollen and healthy microcysts in the DMSO control group 10 days after incubation at 37°C. (B, C) Damaged microcysts after 4 and 7 days of treatment with imatinib at a concentration of 10 *μ*g/mL. Loss of cyst swelling, detachment of the germinal layer away from the laminated layer, degeneration, and disruption of microcyst layers are visible. Note the dense necrotic region at the center of the altered cysts.

The effects of imatinib alone and in combination with albendazole on microcysts were evaluated daily for 15 days using an inverted microscope. During the incubation period, microcysts in the control group maintained a stable appearance, normal swelling, and turgidity, showing no signs of wrinkling, degradation, or collapse of the germinal layer (Figure 3A). Microscopic observations demonstrated that, after a few days of incubation with various drug concentrations, *E. granulosus* microcysts underwent distinct morphological changes. Observed changes included loss of swelling, collapse, germinal layer detachment, and wall degeneration (Figure 3B,C).

In this study, the effects of imatinib alone (at final concentrations of 1, 2.5, 5, 10, and 15 *μ*g/mL) as well as the combination of 10 *μ*g/mL albendazole + 1 *μ*g/mL imatinib and 5 *μ*g/mL albendazole + 1 *μ*g/mL imatinib on microcysts were evaluated.

**Figure 4:**
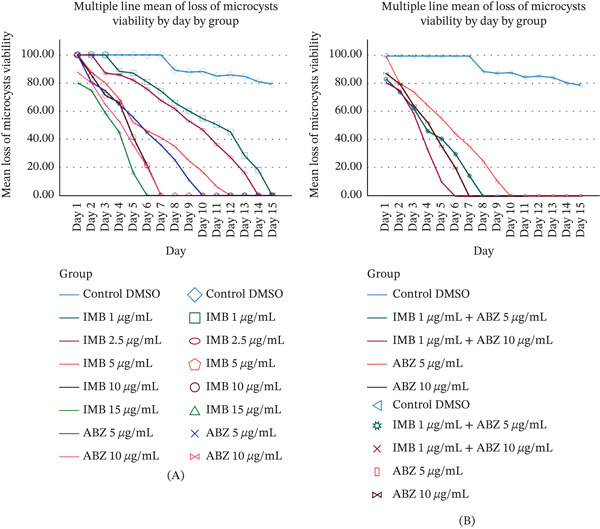
Survival of *Echinococcus granulosus* microcysts during in vitro exposure to (A) imatinib (IMB) alone and to (B) the combination of IMB and albendazole (ABZ). Each point shows the mean proportion of viable microcysts at different time points, obtained from three independent experiments.

The viability of *E. granulosus* microcysts is shown in Figure 4. Among the various concentrations of imatinib, the highest effect on microcysts was observed at 15 *μ*g/mL, whereas the weakest effect was seen at 1 *μ*g/mL, with complete destruction occurring on Days 6 and 15, respectively.

Imatinib at a concentration of 15 *μ*g/mL and the combination of 10 *μ*g/mL albendazole + 1 *μ*g/mL imatinib both led to complete destruction of all microcysts by Day 6 (*p* < 0.05). Imatinib at 10 *μ*g/mL and albendazole at the same concentration exhibited similar potency, resulting in the complete destruction of all microcysts by Day 7. In contrast to the comparable effects of imatinib and albendazole at 10 *μ*g/mL, complete destruction of all microcysts occurred on Day 10 for albendazole at 5 *μ*g/mL and on Day 12 for imatinib at 5 *μ*g/mL.

The combination of 5 *μ*g/mL albendazole + 1 *μ*g/mL imatinib caused complete microcyst destruction by Day 8, whereas at the same time, when imatinib and albendazole were used alone at 5 *μ*g/mL, 34.83*%* ± 2.59*%* and 25.23*%* ± 1.75*%* of the microcysts remained viable, respectively (*p* < 0.05). Similarly, the combination of 10 *μ*g/mL albendazole + 1 *μ*g/mL imatinib achieved complete destruction by Day 6, whereas at the same day, 21.53*%* ± 3.13*%* and 20.36*%* ± 3.56*%* of microcysts remained alive under 10 *μ*g/mL imatinib and 10 *μ*g/mL albendazole alone, respectively. These data further confirm that the combination therapy is significantly more effective than either drug alone (*p* < 0.05).

## 4. Discussion

Recently, imatinib has been shown to be effective against *E. multilocularis*. Imatinib at concentrations of 25 *μ*M (approximately 10 *μ*g/mL) and 10 *μ*M (approximately 4 *μ*g/mL) exhibited significant antiparasitic effects on stem cells, metacestodes, and protoscoleces of *E. multilocularis* under in vitro conditions [13]. In the present study, for the first time, the antiparasitic effects of imatinib alone and in combination with albendazole were evaluated on protoscoleces and microcysts of *E. granulosus*. Our results demonstrated a markedly stronger protoscolicidal effect of imatinib compared with albendazole. Imatinib at both 10‐ and 5‐*μ*g/mL concentrations killed protoscoleces in a significantly shorter period than albendazole at 10 *μ*g/mL (*p* < 0.05). Specifically, imatinib at 10 *μ*g/mL induced approximately twice the effect of albendazole at the same concentration, leading to complete protoscoleces death by Days 15 and 33 postexposure, respectively. Similarly, when comparing imatinib at 5 *μ*g/mL with albendazole at 10 *μ*g/mL, imatinib exhibited superior efficacy, with significant differences observed on days 18,21, and 27, and with all protoscoleces dying on Days 30 and 33, respectively, indicating that even at half the concentration of albendazole, imatinib was more potent. When applied at comparable effect levels, albendazole at 10 *μ*g/mL and imatinib at 2.5 *μ*g/mL both caused complete protoscoleces death by Day 33. In a study by Farhadi et al. [16], similar to the effect of imatinib at 10 *μ*g/mL in the present study, all parasites were eliminated 15 days posttreatment with 10 *μ*g/mL flubendazole‐loaded nanoparticles. In contrast, using free flubendazole at 10 *μ*g/mL over the same period, 44*%* ± 5.22*%* of protoscoleces remained viable. Elissondo et al. [14] also reported a lower protoscolicidal effect of flubendazole under in vitro conditions compared with imatinib at 10 *μ*g/mL used in the present study. Twenty‐five days after exposure to flubendazole, protoscoleces viability decreased to 13.9%, and the maximum effect of flubendazole was observed at 10 *μ*g/mL and on Day 30, achieving 100% mortality of protoscoleces.

Few studies have investigated the effects of imatinib on parasites. In a study conducted by Hemer and Brehm [13], the effect of imatinib on the metacestode (larval stage) of *E. multilocularis* was evaluated over 7 days of incubation at concentrations of 10, 25, and 50 *μ*M. Imatinib at 10 *μ*M prevented the development of metacestode vesicles from parasite stem cells, resulting in a 50% reduction in vesicle formation. At 25 and 50 *μ*M, imatinib exerted a stronger effect on eliminating stem cells, metacestode vesicles, and protoscoleces, leading to complete parasite death after 7 days. *E. multilocularis* larvae possess enzymes with high similarity to human ABL kinases, which have been identified as targets of imatinib in *S*. *mansoni* [17]. In a study by Casado et al. [18] using ivermectin, all protoscoleces were killed within 3 and 5 days after treatment at concentrations of 100 and 10 *μ*g/mL, respectively. In contrast, levamisole at 100 *μ*g/mL caused complete protoscoleces death after 6 days of incubation. In the present study, imatinib at 40 and 10 *μ*g/mL induced complete protoscoleces death on Days 6 and 15, respectively, showing stronger efficacy than levamisole but weaker than ivermectin.

Despite the remarkable efficacy of imatinib in killing protoscoleces compared with albendazole, the combination of imatinib with albendazole produced a stronger and faster effect than imatinib alone. Comparison of imatinib at 10 *μ*g/mL and albendazole at 10 *μ*g/mL with the combination of 5 *μ*g/mL albendazole + 5 *μ*g/mL imatinib (*p* < 0.05), as well as comparison of 20 *μ*g/mL imatinib with the combination of 10 *μ*g/mL albendazole + 10 *μ*g/mL imatinib, showed that the dual‐drug treatment was more effective than either drug alone. Although the number of viable protoscoleces reached zero on Day 12 following treatment with the combination of 5 *μ*g/mL albendazole + 5 *μ*g/mL imatinib (similar to imatinib 20 *μ*g/mL), in the same period, treatment with 10 *μ*g/mL imatinib and 10 *μ*g/mL albendazole resulted in 11*%* ± 3*%* and 71*%* ± 4.1*%* of protoscoleces remaining viable, respectively.

Furthermore, 9 days after exposure of protoscoleces to the combination of 10 *μ*g/mL albendazole + 10 *μ*g/mL imatinib, parasite viability reached zero. In contrast, during the same period, exposure to 10 *μ*g/mL imatinib alone resulted in 36.33*%* ± 5.5*%* viable protoscoleces (*p* < 0.05), and exposure to 10 *μ*g/mL albendazole alone resulted in 79.66*%* ± 4.5*%* viability (*p* < 0.05). These results demonstrate that the combination of 10 *μ*g/mL albendazole + 10 *μ*g/mL imatinib exerts greater protoscolicidal effects than the sum of the individual drug effects. This was particularly evident on Day 12, when the number of viable protoscoleces reached zero. Interestingly, these findings suggest a potential synergistic effect when the two drugs are used in combination, leading to enhanced efficacy compared with monotherapy. Imatinib may exert its effects through inhibition of parasite kinase activity [19, 20] and albendazole primarily disrupts microtubule formation [21, 22]; therefore, their combined use may enhance antiparasitic efficacy through complementary mechanisms, consistent with recent interest in multitarget therapeutic strategies against helminth infections. Several other studies have also shown that combining imatinib with drugs such as albendazole or mebendazole can produce synergistic effects. For example, the combination of mebendazole with imatinib or dasatinib demonstrated significant anticancer activity in both sensitive and resistant CML cells [23–26].

Urrea‐París et al. [27] investigated the effects of drug combinations on protoscoleces. In their study, *E. granulosus* protoscoleces were cultured in vitro with albendazole, praziquantel, or both drugs together. The combined use of praziquantel and albendazole resulted in a faster reduction in protoscoleces viability compared with each drug alone. Despite the low concentrations of albendazole + praziquantel used, protoscoleces viability decreased significantly within 15 days. Similarly, the combination of albendazole and mebendazole nanocapsules exhibited greater efficacy than either drug alone [28]. These findings are consistent with our results, where the combination of imatinib and albendazole showed superior effects on protoscoleces compared with the individual drugs.

In a study, Elissondo et al. [29] investigated the combined effects of ivermectin and flubendazole on protoscoleces. The combination of 1 *μ*g/mL FLBZ + 1 *μ*g/mL IVM resulted in protoscoleces survival of 4.4*%* ± 3*%* after 12 days. In contrast, in the present study, the combination of 5 *μ*g/mL albendazole + 5 *μ*g/mL imatinib led to the death of all protoscoleces after 12 days, and the combination of imatinib and albendazole was more effective in reducing protoscoleces viability than the ivermectin and flubendazole combination, although the drug doses used in their study were lower.

Richter et al. [30] conducted a study to evaluate the effect of triclabendazole and closantel on the metacestode of *E. multilocularis* under in vitro conditions. Triclabendazole at a concentration of 20 *μ*g/mL caused severe damage to the vesicles within 12 days. In contrast, in our results, the use of much lower concentrations and shorter incubation times led to the death of protoscoleces.

In the present study, SEM analysis revealed that imatinib induced significant structural and ultrastructural damage in protoscoleces. Observed ultrastructural alterations included rostellum deformation leading to hook loss, microtrix disappearance, and contraction in the soma region.

Our results demonstrated that, under in vitro conditions, imatinib alone and in combination with albendazole effectively affected *E. granulosus* microcysts (metacestodes), with the highest efficacy observed at a concentration of 15 *μ*g/mL. Loss of cyst swelling was one of the earliest signs of damage, detectable by inverted microscopy as early as Day 2 postincubation. Complete destruction of microcysts was observed by Day 6. In comparison, similar structural degeneration occurred after 18 days of treatment with albendazole‐loaded nanoparticles [31] and within 7 days in 50% of metacestodes treated with ursolic acid [32]. Hemer and Brehm [13] reported that imatinib at concentrations of 50 and 25 *μ*M caused complete death of metacestodes after 7 days, whereas at 10 *μ*M, approximately 50% of metacestodes were killed in the same period. In the present study, imatinib exhibited almost comparable efficacy to albendazole at similar concentrations; after 7 days of exposure to either 10 *μ*g/mL imatinib or 10 *μ*g/mL albendazole, complete cyst destruction was observed. However, imatinib at 5 *μ*g/mL was slightly less effective than albendazole at the same concentration, with complete microcyst destruction occurring 2 day later (Day 12). Therefore, although imatinib at 10 and 5 *μ*g/mL exerted significantly stronger effects on protoscoleces compared with albendazole, this difference was less pronounced for microcysts; at 10 *μ*g/mL, the effect was comparable, and at 5 *μ*g/mL, it was slightly lower. Nevertheless, the combination of imatinib and albendazole appeared more effective than either drug alone in the in vitro treatment of *E. granulosus* microcysts, similar to observations in protoscoleces. The combination of 5 *μ*g/mL albendazole + 1 *μ*g/mL imatinib showed significantly higher efficacy than each drug alone at 5 *μ*g/mL. Similarly, 10 *μ*g/mL albendazole + 1 *μ*g/mL imatinib demonstrated stronger effects compared with either albendazole or imatinib alone at 10 *μ*g/mL (*p* < 0.05). Complete destruction of all microcysts was observed on Day 7 following treatment with either 10 *μ*g/mL imatinib or 10 *μ*g/mL albendazole. In the group treated with 5 *μ*g/mL albendazole + 1 *μ*g/mL imatinib, complete destruction occurred on Day 8, whereas in the 10 *μ*g/mL albendazole + 1 *μ*g/m imatinib combination, it occurred earlier, on Day 6. Similar observations were reported by Farhadi et al. [16], who demonstrated that 5 and 10 *μ*g/mL concentrations of flubendazole‐loaded nanoparticles caused complete microcyst destruction after 9 and 7 days of treatment, respectively. This study has some limitations. All experiments were conducted under in vitro conditions, which may not fully reflect host–parasite interactions in vivo. Additionally, the relatively small number of microcysts in each group and reliance on morphological evaluations may affect the generalizability of the findings. Therefore, further studies using animal models are needed to confirm the efficacy, safety, and optimal use of imatinib alone and in combination with albendazole.

## 5. Conclusion

The findings of this study indicate that imatinib exhibits stronger antiparasitic activity in vitro against *E. granulosus* protoscoleces compared with albendazole, and combining imatinib with albendazole enhances this effect compared with either drug alone. Although imatinib showed effects similar to albendazole on microcysts, the combination of the two drugs consistently provided superior antiparasitic activity. These results suggest that the combination of imatinib with albendazole may offer better efficacy against *E. granulosus* protoscoleces and microcysts. In the present study, the effects of imatinib and its combination with albendazole were evaluated in an in vitro setting. However, to confirm the efficacy of these drugs in vivo and to assess potential side effects, further studies using animal models are necessary. Therefore, it is recommended that animal studies be conducted to evaluate the efficacy and safety of imatinib alone and in combination with albendazole, providing a foundation for the development of novel and effective treatments for hydatid cysts.

## Author Contributions

Zahra Hanifeh, Ali Haniloo, Negin Torabi, and Mehdi Farhadi conceptualized and designed the study. Zahra Hanifeh and Mehdi Farhadi conducted the experiments. All authors contributed to data analysis and interpretation of the results. The manuscript was drafted by Zahra Hanifeh and Mehdi Farhadi.

## Funding

This study was supported by the Zanjan University of Medical Sciences (10.13039/501100008323) (A‐12‐441‐2).

## Disclosure

The authors accept full responsibility for its content. All authors have read and approved the final version of the article.

## Ethics Statement

All procedures of this study were conducted in accordance with the ethical standards approved by the Ethics Committee of Zanjan University of Medical Sciences (Approval No. IR.ZUMS.BLC.1402.044).

## Conflicts of Interest

The authors declare no conflicts of interest.

## Data Availability

The data that support the findings of this study are available from the corresponding author upon reasonable request. Summary data are provided within the manuscript.
